# Supplementary material for: Effect of prenatal DINCH plasticizer exposure on rat offspring testicular function and metabolism
Source: Sci Rep. 2017 Sep 11;7:11072. doi: 10.1038/s41598-017-11325-7 (PMC5593853; doi:10.1038/s41598-017-11325-7)
Supplement: Supplementary file 1 — Supplementary Information [file 41598_2017_11325_MOESM1_ESM.pdf]

Effect of prenatal DINCH plasticizer exposure on rat offspring testicular function and metabolism

Enrico Campioli[1,2], Sunghoon Lee[1,3], Matthew Lau[1,4], Lucas Marques[1,4], and Vassilios Papadopoulos[1,2,3,4,5]*

[1]Research Institute of the McGill University Health Centre, Montréal, Québec, Canada

[2]Department of Medicine, McGill University, Montréal, Québec, Canada

[3]Department of Biochemistry, McGill University, Montréal, Québec, Canada

[4]Department of Pharmacology and Therapeutics, McGill University, Montréal, Québec, Canada

[5]Department of Pharmacology & Pharmaceutical Sciences, School of Pharmacy, University of Southern California, Los Angeles, California, USA

***Corresponding author:** Dr. Vassilios Papadopoulos, Department of Pharmacology and Pharmaceutical Sciences, School of Pharmacy, University of Southern California, Los Angeles, CA 90089, USA; e-mail: vpapadop@usc.edu

Abstract

In 2002, the plasticizer 1,2-cyclohexane dicarboxylic acid diisononyl ester (DINCH) was introduced in the European market as a substitute for endocrine-disrupting phthalates. We found that *in utero* exposure of rats to DINCH from gestational day 14 until parturition affected reproductive organ physiology and reduced circulating testosterone levels at post-natal day 60, indicating a long-term effect on Leydig cells of the testis. Metabolically, animals exhibited randomly increased serum glucose concentrations not associated with impaired glucose utilization. Analysis of liver markers in the serum showed a hepatic effect; e.g. reduced bilirubin levels and albumin/globulin ratio. At post-natal day 200, random appearance of testicular atrophy was noted in exposed offspring, and limited changes in other reproductive parameters were observed. In conclusion, DINCH exposure appears to directly affect Leydig cell function, likely causing premature aging of the testes and impaired liver metabolic capacity. These effects might be attenuated with physiologic aging.

Introduction

Since the first use of rubber around 1600 BC to model artifacts and other objects[1], humans have manipulated natural materials (e.g. rubber, nitrocellulose, cellophane, casein derivates) and developed complex synthetic polymers (e.g. bakelite, polyvinyl chloride [PVC], polystyrene, acrylate polymers, polyethylene, and formica laminates to cite some)[2] in order to improve their quality of life. Despite this longstanding use of natural rubber, it was only 160 years ago that Alexander Parkes created and patented the first man-made synthetic plastic polymer, known as parkesine[3]. In 1872, Eugen Baumann discovered PVC, but it was not until 1907 that Leo Baekeland created bakelite, the first synthetic mass-produced plastic[4]. Bakelite and PVC, as well as many other synthetic polymers, are very hard materials, which can limit their use. The rapid advancement in technology in the 20th century created a number of different challenges for the plastics industry. Predictably, with the mass production of plastics came the use of additives (e.g., inorganic fillers, plasticizers, and fire retardants) to enhance polymer performance[5,6]. Plasticizers are added to increase the flexibility, pliability, and elasticity of plastics[5,6]. In ancient times, water, vegetable and sperm oil were used as natural plasticizers to soften natural materials (e.g. clay, leather, resins, rubber)[2] into more ductile materials[7]. However, water and oil are not suitable for use in modern, mass-produced plastics. In plastics such as PVC, plasticizers are only dispersed into the polymer due to the lack of covalent bonds[6]; therefore, they are readily released into the environment, which can lead to human and animal exposure through ingestion[6,8], dermal contact[8], and/or inhalation[9]. Thus, exposure to these compounds is of concern due to potential adverse health effects.

Most plasticizers currently used in the plastics industry are phthalates, in particular, 2-ethylhexyl phthalate (DEHP) which, along with its metabolites, has been shown to affect the testis with its anti-androgenic proprieties[6,10]. The testis is organized in two compartments[11] (i) the seminiferous tubules surrounded by myoid peritubular cells and containing germ cells and supporting somatic Sertoli cells, and (ii) the interstitium comprising the endocrine Leydig cells responsible for the production of testosterone, blood vessels, mesenchymal and various hematopoietic cells, such as macrophages promoting inflammatory

responses and protection against infection[41]. Studies in a variety of animal models have shown that DEHP affects testosterone production and Leydig cell homeostasis and induces cryptorchidism[6,12]. The finding of a strong correlation between DEHP and several types of cancer[12], as well as adverse effects on the female reproductive system (e.g., premature breast development, decrease in litter size, and risk of mid-pregnancy abortion in murine models)[6,12,13], highlighted the need for an alternative plasticizing agent.

In 2002, 1,2-cyclohexane dicarboxylic acid diisononyl ester (DINCH) was introduced as an alternative to DEHP. Although DEHP is still widely used in some medical applications, such as the production of blood bags[14], DINCH received final approval in 2006 for use in articles intended to contact food in Europe, Switzerland, Australia, Japan, Korea, Taiwan, and China[15,16]. However, the effect of DINCH exposure on human health remains unclear, despite continued study by many research groups. According to a 2006 European Food Safety Authority report[17], DINCH is not genotoxic but does exhibit sex-specific renal and thyroid toxicity at high doses in rats (300–1000 mg/kg body weight/day). No evidence of developmental or reproductive toxicity was observed in prenatal and two-generation toxicity studies in Wistar rats and rabbits at doses of up to 1000 mg/kg body weight/day[16]. Furr et al. administered 750 mg/kg/day of DINCH to pregnant rat dams from gestational day (GD) 14 through GD 18, with no adverse effect on fetal testosterone production[18]. We recently reported that a possible DINCH metabolite, cyclohexane-1,2-dicarboxylic acid monoisononyl ester (MINCH), affects the differentiation of primary rat preadipocytes present in the stromal vascular fraction through a peroxisome proliferator-activated receptor (PPAR-α)–mediated mechanism[17]. Minguez-Alarcon et al. found a negative association between urinary concentrations of MHiNCH, a DINCH metabolite, and total oocyte yield in women ≥37 years old[19]. Using a TM4 Sertoli cell model, Nardelli et al. demonstrated that DINCH differentially regulates as many as 648 different genes involved in processes including cellular movement, glutathione-mediated detoxification, and other signaling pathways (e.g., Ras homolog gene family, member A extracellular signal-regulated kinase/Mitogen-Activated Protein Kinase)[20]. The authors concluded that DINCH is biologically active, as the activity against TM4 cells was higher than that of the positive control, MEHP[20]. In addition, Eljezi et

al. recently reported that both DINCH and DEHP were cytotoxic to L929 murine cells exposed to 0.1 mg/ml of either compound for 7 days[21].

Considering the limited data regarding the effects of DINCH and the ongoing debate over its safety[22,23], we evaluated the potential reproductive effects of exposure to DINCH during a critical developmental window. Pregnant rats were exposed to DINCH, and the effects on their male progeny were then characterized.

Results

1. *In utero* exposure to DINCH alters gene expression on post-natal day (PND) 3 without affecting fetal testosterone production

We measured the weight, anogenital distance (AGD), fetal testosterone level, and testes-specific gene expression of PND 3 progeny exposed *in utero* to DINCH. Based on previous studies of DEHP and testicular function, expression of the following genes was analyzed[24,25]: steroidogenic pathway genes luteinizing hormone/choriogonadotropin receptor (*Lhcgr*), steroidogenic acute regulatory protein (*Star*), translocator protein (*Tspo)*, cytochrome P450 11a1 (*Cyp11a1*), Leydig cell marker platelet-derived growth factor receptor 1a (*Pdgfra*), Leydig/Sertoli cell marker Nestin (*Nes*), Sertoli cell marker Wilms tumor protein (*Wt1*), global testicular marker androgen receptor (*Ar*), thyroid receptor expressed in the testes (*Thra1*), urogenital tract gene insulin like 3 (*Insl3*), proliferation marker proliferation cell nuclear antigen (*Pcna*), and germ cell markers heat shock protein 90 (*Hsp90*) and octamer-binding transcription factor 4 (*Oct4*).

The pups exhibited no significant changes in weight, AGD (Fig. 1A), or fetal testosterone production (Fig. 1B). Quantitative reverse transcription–polymerase chain reaction (qRT-PCR) analyses of gene expression showed a significant 1.63-fold increase in *Cyp11a1* levels at a dose of 100 mg DINCH/kg/day ($p<0.01$, n=10, Fig.1C) compared with controls. Interestingly, *Nes* levels decreased at all DINCH doses tested: 1.32-fold at 1 mg/kg/day ($p<0.01$, n=10), 1.27-fold at 10 mg/kg/day ($p<0.05$, n=9), and 1.27-fold at 100 mg/kg/day ($p<0.05$, n=10, Fig. 1C).

2. *In utero* exposure to DINCH affects reproductive health and metabolic parameters

Considering the reported effects of DINCH exposure on liver and kidney function[15], we examined various parameters associated with reproductive health and metabolism: AGD, seminal vesicle weight, testicular weight, fat pad weight, and overall weight. Additionally, random glucose, fasting glucose, and serum glucose levels after a glucose tolerance test were measured by the McGill Comparative Medicine and

Animal Resources Centre Laboratory. Finally, serum C-peptide and glycated hemoglobin levels were measured using enzyme-linked immunosorbent assays (ELISAs).

At PND 60, a significant increase ($p<0.05$) in AGD was observed for a dose of 1 mg DINCH/kg/day (10.80 ± 0.29 mm/100 g body weight [bw], n=9), which progressively decreased for DINCH doses of 10 (10.31 ± 0.40 mm/100 g bw, n=8) and 100 mg/kg/day (9.91 ± 0.24 mm/100 g bw, n=10), compared with controls (9.50 ± 0.32 mm/100 g bw, n=10) (Fig. 2A). Seminal vesicle weight progressively decreased ($p<0.01$) at 1 (0.18 ± 0.01 g/100 g bw, n=9), 10 (0.17 ± 0.01 g/100 g bw, n=8) and 100 (0.13 ± 0.01 g/100 g bw, n=10) mg DINCH/kg/day compared with controls (0.19 ± 0.01 g/100 g bw, n=10). The change with the 100 mg DINCH/kg/day dose alone was significant according to the results of a *post hoc* test ($p<0.01$) (Fig. 2A). DINCH exposure had no effect on fat pad weight, and total weight (Fig. 2A).

Interestingly, random glucose levels increased dramatically at a dose of 1 mg DINCH/kg/day (17.11 ± 1.22 mmol/L, n=15, $p<0.001$) compared with controls (11.99 ± 0.85 mmol/L, n=16), suggesting a possible effect of DINCH on metabolic function (Fig. 2B). Given the consistent effect on random glucose levels, we evaluated fasting glucose, glucose after a glucose tolerance test, C-peptide, and glycated hemoglobin levels, as each of these markers is an indicator of glucose impairment and diabetes (Fig. 2C–F). The results were inconclusive, suggesting that *in utero* exposure to DINCH does not significantly affect metabolism.

At PND 200, a significant increase ($p<0.01$) in overall seminal vesicle weight was observed at DINCH doses of 1 (0.17 ± 0.01 g/100 g bw, n=11), 10 (0.17 ± 0.01 g/100 g bw, n=7) and 100 (0.13 ± 0.01 g/100 g bw, n=11) mg/kg/day compared with controls (0.15 ± 0.01 g/100 g bw, n=9) (Fig. 2G). DINCH had no effect on total weight, AGD, fat pad weight, or testicular weight (Fig. 2G). In addition, no significant changes in random glucose, fasting glucose, glucose after glucose tolerance test, C-peptide, or glycated hemoglobin levels were observed at PND 200 (Fig. 2H–L).

Serum markers of liver and kidney dysfunction were also analyzed. At PND 60, DINCH-exposed animals exhibited a significant decline in magnesium levels, a kidney marker, versus controls (1.69 ± 0.05 mmol/L, n=16) (p<0.0001 the overall value for all doses), and in particular at a dose of 100 mg/kg/day (1.43 ± 0.04 mmol/L, p<0.001, n=16) (Table 1). Regarding markers of liver dysfunction, we observed a significant increase in the albumin/globulin ratio (p=0.012), particularly at a dose of 100 mg DINCH/kg/day (1.21 ± 0.03, p<0.01, n=16), compared with controls (1.07 ± 0.02, n=16) (Table 1). By contrast, total bilirubin levels decreased in a cubic non-monotonic manner (p=0.0009) at doses of 1 mg DINCH/kg/day (8.00 ± 0.44 mmol/L, p<0.001, n=15) and 100 mg/kg/day (8.25 ± 1.01 mmol/L, p<0.01, n=16), compared with controls (12.64 ± 0.83 mmol/L, n=16) (Table 1). Total protein decreased (p=0.0320) at doses of 1 mg DINCH/kg/day (66.67 ± 0.75 g/L, p<0.05, n=15) and 100 mg/kg/day (67.56 ± 1.54 g/L, p<0.05, n=16), compared with controls (73.50 ± 2.47 g/L, n=16) (Table 1). Finally, total triglycerides decreased significantly (p=0.043) at a dose of 1 mg DINCH/kg/day (2.15 ± 0.13 mmol/L, p<0.05, n=15), compared with controls (2.93 ± 0.20 mmol/L, n=16) (Table 1). These effects were not observed in the PND 200 animals (Table 2). In addition, analyses of the blood of the pregnant dams at GD 21 showed no significant changes except for a significant decline in magnesium levels (p=0.0433) at a dose of 10 mg DINCH/kg/day (0.92 ± 0.03 mmol/L, p<0.05, n=16), compared with controls (1.00 ± 0.02 mmol/L, n=16) (Supplementary Table S1). Levels of thyroid-associated hormones (thyroid-stimulating hormone [TSH], triiodothyronine, and thyroxine) were not significantly affected, based on analyses of the combined cohort values, even though some random seasonal differences were observed in certain cohorts (Supplementary Figure S1).

3 *In utero* exposure to DINCH affects circulating testosterone levels, testicular gene markers, and testicular morphology

Testicular gene expression was analyzed in PND 60 and PND 200 progeny exposed *in utero* to DINCH based on previous reports indicating that DEHP exposure affects testicular function[24,25]. The following

genes were examined: steroidogenic pathway markers (*Lhcgr, Star, Tspo, Cyp11a1, Hsd3b*), Leydig cell marker (*Pdgfra*), Leydig/Sertoli cell marker (*Nes*), Sertoli cell markers (*Abp*, *Wt1*), global testicular marker (*Ar*), urogenital tract marker (*Insl3*), cell proliferation marker (*Pcna*), and germ cell markers promyelocytic leukaemia zinc finger (*Plzf*), *Hsp90* and *Oct4*. Following gene expression analysis, levels of plasma testosterone and serum luteinizing hormone (LH) were determined, and proinflammatory activity was assessed via immunohistochemical analysis of tissue sections for CD68 expression.

At PND 60, we observed significant increase in the expression of genes involved in steroidogenesis (particularly *Star* and *Cyp11a1*) at a dose of 1 mg/kg/day (respectively 1.72- and 1.69-fold, n=13, p<0.05), compared with controls. In addition, a significant change in the global expression of *Tspo* was observed at all doses (p<0.05) (Fig. 3A). Interestingly, the expression of *Ar* was reduced at all DINCH doses (p<0.05), compared with controls, whereas DINCH significantly affected the expression of *Plzf* at a dose of 10 mg/kg/day (28% decrease, p<0.05), and tended to suppress *Plzf* expression at the other doses, although the differences were not significant (Fig. 3A).

A cubic non-monotonic effect (p=0.0029) resembling a typical hormonal profile was observed with plasma testosterone levels. Plasma testosterone levels declined significantly at doses of 1 mg DINCH/kg/day (1.37 ± 0.17 ng/ml of plasma, n=15, p<0.01) and 100 mg/kg/day (1.23 ± 0.18 ng/ml of plasma, n=15, p<0.01), compared with controls (2.48 ± 0.33 ng/ml of plasma, n=15) (Fig. 3B). To exclude a possible central effect, serum LH was measured by ELISA, but no significant changes were observed (Fig. 3C). Finally, to examine the possibility of a window-specific effect, testosterone levels were measured in a different cohort of animals exposed to DINCH (100 mg/kg/day) from GD 8 until parturition. The results showed that exposing the animals earlier in development (and therefore for a longer period) abrogates the effect on testosterone production, suggesting that the animals can compensate for the biological effects of the plasticizer (Fig. 3D).

Morphological analyses of testicular tissue sections by Periodic acid–Schiff (PAS) revealed an increased occurrence of enlarged interstitial spaces in the DINCH-treated rats at PND 60 (Fig. 3E). The staining with anti-collagen 1 revealed that this isoform is present in the interstitial space of every sample including the enlarged interstitial spaces (Fig. 3F). Therefore, we postulated that an inflammatory activation had occurred. Since the deregulation of the tissue resident macrophages it is known to be the cause of inflammatory and fibrotic events[26], which are in turn a cause of possible decrease of testosterone production[27], leading to testicular aging, we decided to stain the sections for CD68, a well-known macrophage marker. The results showed increased expression of the macrophage marker in the interstitial space ($p<0.05$), particularly at the dose of 1 and 100 mg DINCH/kg/day (1.83-fold and 1.99-fold respectively, n=6, $p<0.05$) compared to controls (Fig. 4).

In PND 200 offspring, a significant increase in *Pdgfra* was observed only for a dose of 10 mg DINCH/kg/day (1.76-fold, n=7, $p<0.05$), compared with controls, and a significant change in global expression of *Cyp11a1* was observed at all doses (cubic non-monotonic p=0.019), with a significant peak in animals exposed to the 1 mg/kg/day dose (2.26-fold, n=11, $p<0.05$) (Fig. 5A). Plasma testosterone exhibited a quadratic non-monotonic effect (p=0.0302), with no dose producing a significant effect (Fig. 5B) and no changes in serum LH levels (Fig. 5C). The decline in the effect of DINCH exposure on gene expression and hormonal stimulation can be explained by the aging of the animals, which could mask the biological effects. Moreover, the observed effect of DINCH exposure on increasing the size of interstitial spaces and CD68 expression at PND 60 was lost in PND 200 animals (Fig. 5D and 5E). This observation suggests that the effect of DINCH could be related to premature aging of the testis. It is noteworthy that of 13 control animals and animals exposed to 100 mg DINCH/kg/day, one of the control animals and three of the DINCH-treated animals exhibited testicular atrophy (Fig. 6A). Interestingly, the PAS staining (Fig. 6B) and CD68 immunostaining (Fig. 6C) revealed the modified architecture of the atrophic testes. The seminiferous tubules are smaller, with severe loss of germ cells; the interstitial space is increased and the

interstitial cells seemed clustered together. These data suggest that DINCH exposure could be particularly damaging to the testes, warranting further investigation.

Discussion

Plastic polymers that have been enhanced by the use of plasticizers have become an essential part of our lives. Plastics cannot be completely replaced because they are a central part of the economic and technological aspects of modern human society. Nonetheless, concerns over potential health and environmental effects of phthalates and other plasticizers have been raised in the scientific community since the 1940s[28], and these compounds have been the focus of thousands of studies performed to evaluate potential harmful effects. If we expand the concept of Ludwig Feuerbach, "*Man is what he eats*", to "*Man is what he eats and where he lives*", it becomes more clear how studying the potential health effects of plastics and plasticizers is necessary in order to avoid long-term consequences that can impact the health and wellbeing of future generations. Unfortunately, even though DINCH was introduced in the market in 2006 for applications involving food contact, the scientific community still lacks sufficient information on its biological safety.

Many studies have reported an association between phthalate exposure and reproductive health parameters. Dalsenter et al. reported that *in utero* and lactational exposure of Wistar rats to DEHP at 500 mg/kg/day reduces ventral prostate and seminal vesicle weight and impairs spermatogenic processes[29]. We showed that *in utero* exposure from GD 14 until parturition to DEHP at concentrations ranging from 234 to 1250 mg/kg/day induces an increase in the absolute volume of Leydig cells, which is correlated with low serum testosterone levels[30]. Noriega et al. administered DEHP from GD 23 until GD 56 or GD 98 to pubertal Sprague-Dawley and Long-Evans rats[31]. They found that at high doses of 300 and 900 mg/kg/day, DEHP delayed the onset of puberty and reduced androgen-dependent tissue weights, especially in Long-Evans rats. By contrast, the Sprague-Dawley rats exhibited more severe changes in testis weight and testicular histology, as well as epididymal weight and sperm counts starting at midpuberty[31]. Overall, after DEHP treatment, testosterone release was reduced *in vivo*, and the results were confirmed in *ex vivo* experiments[31]. Interestingly, *in utero* exposure of mice from GD 7 until GD 14 to DEHP at a dose of 150 mg/kg was shown to cause a decrease in seminal vesicle weight in the F3

progeny, suggesting a transgenerational effect of DEHP[32]. For DINCH, however, the relatively limited information available on reproductive effects shows that exposure of pregnant Wistar rats to a high dose of 1200 mg/kg/day from GD 6 to GD 19 causes maternal and fetal toxicity[15]. Even though other studies performed by the company BASF have reported random unrelated effects on testicular and reproductive function[15], no systemic peer-reviewed studies have been conducted to assess the safety of DINCH. The purpose of the present work, therefore, was to evaluate the effect of DINCH on male progeny exposed *in utero* from GD 14 until birth.

After *in utero* exposure to DINCH, we observed a decrease in *Nes* expression at PND 3, and this decrease was not correlated with a decrease in fetal testosterone production or change in AGD. The absence of an effect on testosterone production agreed with the study of Furr et al., who found no significant change in fetal testosterone production after administration of 750 mg/kg/day of DINCH to pregnant rat dams from GD 14 to GD 18[18]. We also detected a significant change in the expression of the steroidogenic enzyme gene *Cyp11a1*, which metabolizes cholesterol to pregnenolone (the first steroid in the pathway) at a dose of 100 mg DINCH/kg/day. We explain these premature changes in the expression of genes involved in testicular function as an initial sign of a "perturbed" testicular state. The results of analyses of tissues from the PND 60 and PND 200 progeny were indicative of an even more serious and pronounced effect on testicular function. In PND 60 animals, we observed a non-monotonic response in circulating testosterone levels in progeny of animals exposed to doses of 1 and 100 mg DINCH/kg/day, and we observed random fluctuations in the expression of genes critical in androgen formation (*Star, Tspo*, *Cyp11a1*), mechanism of action of androgen (*Ar*) and required in adult male germ cells for stem cell self-renewal (*Plzf*) at different DINCH doses. The PND 60 animals also exhibited a decrease in seminal vesicle weight and changes in the AGD. The changes in testosterone production were not correlated with variations in LH levels, thus suggesting a local testicular effect of DINCH. It should be noted that when the window of exposure was changed from GD 14–birth to GD 8–birth, the effect of DINCH on testosterone production at a dose of 100 mg/kg/day was lost. GD 14 is the point at which the male reproductive system

differentiates and develops, whereas GD 8 is the point at which organogenesis begins. This finding suggests that although DINCH exposure affects the male reproductive system when the exposure occurs during the sensitive window for reproductive development, compensatory mechanisms seem to allow for adaptation of the developmental process when exposure begins earlier, such as at the beginning of organogenesis. Such adaptation allows the embryo to survive and develop.

By PND 200, the effect of DINCH on gene expression in the progeny was lost, except for the steroidogenic marker *Cyp11a1* and the Leydig cell marker *Pdgfra*. Additionally, testosterone production did not change at the 1 and 100 mg/kg/day of DINCH, as was the case with the PND 60 progeny, but only the overall DINCH treatment reached significance in the analysis of variance (ANOVA) test. These changes in the levels of circulating testosterone can be explained by the well-defined changes in testosterone levels associated with aging. Indeed, a comparison of the control rats at PND 60 and PND 200 revealed a 34% reduction in circulating testosterone levels at PND 200 compared with PND 60. In some cases, a reduction in testosterone level in aging rats is associated with reduced LH stimulation[33]. In fact, the observed 34% decrease in testosterone levels was accompanied by a 37% decrease in serum LH levels, suggesting that aging can mask the effect of DINCH. Moreover, no difference was observed in AGD between PND 200 control and DINCH-exposed animals, and only the overall ANOVA value was significant for seminal vesicle weight. This difference between PND 60 and PND 200 could also be explained by aging and associated general weight gain. Despite this loss of activity likely due to aging, it is interesting to note that the initial *in utero* perturbation of testicular function by DINCH can be seen in the adult offspring through changes induced in gene expression, thus corroborating the hypothesis of a direct effect of DINCH on testicular functions. The well-described effect of phthalate plasticizers on Leydig cell function[6,10,12], the testis gene expression changes observed in response to in utero exposure to DINCH (Fig. 1C, 3A and 5A), and the recent finding that DINCH affects Leydig cell function in vitro[34] provided the rational to focus the scope of this paper on the endocrine component of the testis, the Leydig cell.

Interestingly, at PND 200 we observed an increased presence of testicular atrophy in progeny from animals exposed to 100 mg DINCH/kg/day. Albert et al. recently reported that *in utero* exposure to DINCH from GD 8 to PND 21 led to the formation of hemorrhagic testes at the dose of 30 and 300 mg/kg/day[35]. This finding with a different exposure time compared with those used in the present study could confirm a potential endocrine-disrupting pattern that warrants further investigation.

Analysis of our overall results indicates that the exposure window is critical with regard to the effect on testicular development and function. An explanation for the DINCH activity can be found in the biology of rat adult Leydig cells (ALCs), which are present in pup testes as stem Leydig cells (SLCs) that undergo a four-stage developmental process to become ALCs[36]. In the first stage, the SLCs are positive for PDGFRA and nestin but negative for steroidogenic markers unless stimulated *in vitro*[37]. Around PND 21, the second phase begins, and the SLCs become progenitor Leydig cells (PLCs) and begin expressing LH receptor as well as other steroidogenic markers, such as 3-α-hydroxysteroid dehydrogenase (3α-HSD), and 3β-HSD[36]. The third stage, or maturation, begins around PND 28, at which time the PLCs become immature Leydig cells, which are characterized by morphologic changes (e.g., expansion of the endoplasmic reticulum and deposition of lipids) and increased production of steroidogenic enzymes such as CYP11A1, which regulates testosterone production in ALCs. Nestin is an important protein expressed in SLCs of fetal and adult testes, as well as in the central and peripheral nervous system and other endocrine organs[38-41]. An inverse relationship exists in the testes between nestin expression and maturation into ALCs'; for this reason, nestin can be considered a specific SLC marker. Our data clearly demonstrate that DINCH significantly affects this sensitive marker and suggest that in the GD 14–birth window of exposure, DINCH targets the ALC differentiation process. A consequence of disruption of ALC maturation is reduced testosterone production in the young adult offspring, which appears to recover, though this is superficial due to aging, which is associated with a physiologic decline in androgen production.

Nearly 20 years ago, Schultz et al. reported that PPAR-α is present in developing and adult testes in both rats and humans[42]. Human testicular Leydig cells and tubular cells express PPAR-α, which affects their growth and differentiation[42]. Using an *in vitro* model of primary pre-adipocytes (stromal vascular fraction), we previously found that a DINCH metabolite, MINCH, exerts activity via a PPAR-α–mediated mechanism[7]. Moreover, preliminary data from the present study (not shown) showed increased expression of PPAR-α in the liver at PND 60. Images of PAS-stained tissue sections revealed an increase in the random occurrence of fibrotic areas in the testes. Other studies have correlated inflammation with fibrosis and infertility[43,44]. Also, it is well-established that macrophages offer an important protection mechanism for the developing germ cells in the testes[45]. In macrophages, PPARs are involved in both cholesterol and fatty acids metabolism in humans and mice[46] and inflammatory processes[47]. Immunohistochemical analyses of the inflammatory marker CD68 revealed an increased presence of macrophages in the interstitial areas of PND60 animals, suggesting a possible activation of the inflammatory pathway, which has been proposed to lead to fibrosis[26]. This result correlated well with our previous research on DEHP and inflammation in adipose tissue[48] and with other reports that describe proinflammatory effects of phthalates in the testes[49-51]. Interestingly at PND200 the effect was lost, suggesting that age-related changes in the testes affect susceptibility to DINCH.

A study conducted by BASF and reported by Bhat et al. described reduced serum bilirubin levels in female Wistar rats exposed for 13 weeks to low doses of dietary DINCH[15]. The effect was significant at day 30 of treatment but progressively disappeared with prolonged exposure, suggesting a possible compensatory effect in the liver[15]. In our study, changes in bilirubin levels were observed in the PND 60 progeny but not in dams exposed acutely to the compound, an effect that declined at PND 200. Both Bhat et al. and a report from the European Food Safety Authority confirmed that the major organs affected by chronic DINCH exposure are the liver, kidney, and thyroid[15,16]. In the liver of male and female Wistar rats, chronic exposure to mid to high doses of DINCH increased organ weight without changes in general body

weight or hepatic histopathology[15,16]. In the same study, the thyroid appears to play the major role with respect to hepatic toxicity due to a compensatory mechanism involving prolonged metabolic enzyme induction[15]. Our results concerning levels of the major hormones regulating thyroid function did not show any significant changes after acute exposure to DINCH, either in dams or their PND 3 pups. However, it is important to note the evidence of seasonal variations in TSH levels in some DINCH-exposed cohorts. The changes in the averaged cohort-values were not significant, however. This is not surprising in light of previous literature on DINCH. In fact, only a 13 week exposure to 1500, 4500 or 15000 ppm of DINCH in Wistar rats, resulted in modified thyroid weight and altered level of TSH[15,16].

Analysis of the blood of pregnant dams showed significant changes in magnesium levels, whereas the PND 60 progeny exhibited the aforementioned decreases in total bilirubin, total protein, the albumin/globulin ratio (as a liver marker), and magnesium (as a kidney marker), along with a dose-specific decrease in triglycerides (1 mg DINCH/kg/day). The major metabolic observation during the study was the 43% increase compared with controls in random glucose concentrations in the PND 60 progeny of rats exposed to 1 mg DINCH/kg/day. Studies conducted on Wistar rats involving oral administration of increasing doses of DEHP from GD 8 to GD 21 showed high blood glucose, low serum insulin levels and impaired glucose and insulin tolerance in the PND 60 progeny, possibly resulting from epigenetic changes[52]. Zhang et al. reported that chronic exposure of adult male Sprague-Dawley rats to DEHP results in an increased blood glucose and insulin concentrations, leading to insulin and glucose sensitivity[53]. Additionally, through an *in vitro* experiment, they linked the changed phenotype to a PPAR-γ–mediated mechanism[53]. Due to the results obtained in the blood analyses in the present study, we conducted a glucose tolerance test and examined markers of glucose impairment (fasting glucose, C-peptide, and glycated hemoglobin). The results were inconclusive for all three tests for both PND 60 and PND 200 animals, however. Furthermore, as previously described for the reproductive parameters, the effect of DINCH on random glucose is lost at PND 200, possibly due to aging. Thus, additional studies are required to elucidate the underlying cause of increased glucose levels and their significance. This is

critical due to the observed changes in liver markers (albumin/globulin ratio, total bilirubin and total protein levels) at PND 60 and because of the known liver toxicity of DINCH[15,16].

In conclusion, our results indicate that *in utero* exposure to DINCH affects ALC function, causing premature aging of the testes. This results in decreased androgen production, physical changes to the seminal vesicles and AGD, as well as the occurrence of testicular atrophy, which are signs of aging. These reproductive changes are followed by a marked effect on random glucose levels and impaired liver metabolic capacity. These outcomes appear to be attenuated with the physiologic aging of the animal. Further studies are essential to determine the long term consequences of DINCH exposure, and in particular its capacity to induce premature aging of the male reproductive system.

Methods

Chemicals. DINCH was a gift from Hanno Erythropel, Richard Leask, and Milan Maric (McGill University, Montreal, QC, Canada; product number 51303880, "HEXAMOLL DINCH", batch BASFDE).

Animals. Timed pregnant Sprague-Dawley rats were purchased from Charles River Laboratories (Saint-Constant, QC, Canada) and gavaged daily with corn oil or 1, 10, or 100 mg of DINCH/kg/day from GD 14 until parturition (corresponding to PND 0) or from GD 8 until parturition. Pregnant dams were weighed every 2 days, and doses were adjusted accordingly. Male offspring were euthanized at PND 3 or PND 60, and tissues were collected and snap-frozen in liquid nitrogen or fixed in 4% paraformaldehyde (MJS BioLynx Inc., Brockville, ON, Canada). Animals were handled according to protocols approved by the McGill University Animal Care and Use Committee. We chose 1, 10, and 100 mg DINCH/kg/day for comparison with previous experiments we conducted on the phthalate DEHP.

Testis Organ Culture. The testes from PND 3 pups from the control and 100 mg DINCH kg/day groups were cut into 4–10 pieces and placed onto a 7-mm diameter sterile mesh (one testis per mesh). The meshes were then placed inside Millicell culture inserts (Millipore, Billerica, MA, USA) in 24-well plates, with each well containing 300 µl of Dulbecco's modified Eagle's medium/Ham's F12 (1:1) GlutaMax (Gibco) and 80 mg/ml of gentamycin (Sigma-Aldrich). Samples were incubated for 24 h (day 1), and then the medium was collected and replaced with either control medium to assess basal steroidogenesis or with 50 ng/ml of human chorionic gonadotropin (hCG) to test Leydig cell hormone responsiveness. After 24 h (day 2), the medium was collected and the testosterone concentration was determined by radioimmunoassay (RIA).

Blood Collection, Serum Analysis, and Glucose Tolerance Test. Blood was collected by cardiac puncture (PND 60, PND 200), decapitation (PND 3), or through the jugular vein (pregnant dams, glucose tolerance animals) per experimental needs in specific serum tubes (BD Bioscience). Serum was separated

via centrifugation at 2000 × *g* for 15 min and sent to the McGill Comparative Medicine and Animal Resources Centre Laboratory for analysis or used for ELISA. Plasma was collected in EDTA tubes, separated via centrifugation at 2000 × *g* for 15 min, and used for RIA experiments. For glucose tolerance testing, an oral glucose tolerance test was performed on PND 60 animals, and an intraperitoneal glucose tolerance test was performed on PND 60 animals. For both treatments, glucose solution was administered by oral gavage or intraperitoneally at a dose of 2 g/kg (Sigma-Aldrich).

Testosterone RIA. Testosterone levels in the plasma were measured by RIA (MP Biomedical) as previously described[30].

Quantitative Reverse Transcription–Polymerase Chain Reaction (qRT-PCR). Total RNA was extracted from testes and kidney samples using an RNeasy PLUS Mini kit (Qiagen Inc., Mississauga, ON, Canada), and cDNA was prepared using a Transcriptor First-Strand cDNA Synthesis kit (Roche Applied Science) according to the manufacturer's instructions[48,54]. The resulting cDNAs were diluted with nuclease-free water and subjected to qRT-PCR using the SYBR green dye technique on a Light Cycler system 480 (Roche Applied Science) as previously described[48,54]. The results for each RNA product were normalized to β-actin mRNA (*Actb*) to correct for differences in the amount of template cDNA. The oligonucleotide sequences of the sense and antisense primers are shown in Supplementary Table S2.

ELISA. ELISAs were used to quantify serum levels of rat C-peptide (Mercordia AB, Uppsala, Sweden), rat glycated hemoglobin (Neo Scientific, Cambridge, MA, USA), and rat LH (Cusabio, Wuhan, Hubei, China) according to the manufacturers' instructions. Absorbance was read at 450 nm using a VICTOR™ X5 Multilabel Plate Reader (PerkinElmer Inc., Waltham, MA, USA).

PAS staining and Immunohistochemistry (IHC). Tissue fixation and sectioning for PAS staining and IHC were performed using the Research Institute of the McGill University Health CentreHistopathology

Platform. IHC was performed as previously described[25]. Briefly, sections of testes (4 μm) were dewaxed and rehydrated with Trilogy solution (Cell Marque, Rocklin, CA, USA), and antigen was retrieved using DAKO solution (Agilent Technologies Inc. Santa Clara, CA, USA). Incubation with anti-CD68 primary antibody (1:100 dilutions, Abcam, Cambridge, MA, USA) or anti-collagen 1 primary antibody (1:200 dilutions, Abbiotec, San Diego, CA, USA) was conducted overnight at 4°C, followed the next day by a 1-h incubation with the secondary antibody, biotin-labeled goat anti–mouse Ig (1:100, BD Biosciences, San Jose, CA, USA). Slides were developed with streptavidin-horseradish peroxidase solution (Life Technologies, ThermoFisher Scientific, Burlington, ON, Canada), counterstained with hematoxylin (Life Technologies), and finally mounted with CLEAR-MOUNT Tris buffer solution (Electron Microscopy Sciences, Hatfield, PA, USA). Macrophages stained with CD68 were quantified using the software Image Pro-Plus (Media Cybernetics, Rockville, MD, USA). The normalized results are expressed in counts per number of tubules and averaged between the different areas of the photographed testis.

Statistical analyses. Data are expressed as the mean ± standard error of the mean (SEM) and were analyzed using one-way or two-way ANOVA followed by Dunnett's post hoc tests or t-tests using the GraphPad Prism program (GraphPad Software, La Jolla, CA, USA).

Reference List

1 Hosler, D., Burkett, S. L. & Tarkanian, M. J. Prehistoric polymers: rubber processing in ancient mesoamerica. *Science* **284**, 1988-1991 (1999).

2 PlasticsEurope. *What is Plastic?* http://www.plasticseurope.org/what-is-plastic.aspx (2017).

3 UK_Patent_Office. in *Patents for inventions. Abridgments of specifications* (ed UK Patent Office) 255-256 (1865).

4 American_Chemical_Society_National_Historic_Chemical_Landmarks. in *Bakelite: The World's First Synthetic Plastic* (1993).

5 Andrady, A. L. & Neal, M. A. Applications and societal benefits of plastics. *Philos. Trans. R. Soc. Lond B Biol. Sci* **364**, 1977-1984, doi:10.1098/rstb.2008.0304 (2009).

6 Halden, R. U. Plastics and health risks. *Annu. Rev. Public Health* **31**, 179-194, doi:10.1146/annurev.publhealth.012809.103714 (2010).

7 Seymour, R. B. & Rudolph, D. D. in *History of Polymeric Composites* (ed VNU Science) (1987).

8 Frederiksen, H., Skakkebaek, N. E. & Andersson, A. M. Metabolism of phthalates in humans. *Mol. Nutr. Food Res* **51**, 899-911, doi:10.1002/mnfr.200600243 (2007).

9 Wilson, N. K., Chuang, J. C., Morgan, M. K., Lordo, R. A. & Sheldon, L. S. An observational study of the potential exposures of preschool children to pentachlorophenol, bisphenol-A, and nonylphenol at home and daycare. *Environ. Res* **103**, 9-20, doi:10.1016/j.envres.2006.04.006 (2007).

10 [Substance monograph for phthalates - New and updated reference and oxidized monoester metabolites in urine of children-and adults. Opinion of the Commission "Human-Biomonitoring" by the Federal Environment Agency]. *Bundesgesundheitsblatt. Gesundheitsforschung. Gesundheitsschutz* **54**, 770-785, doi:10.1007/s00103-011-1278-1 (2011).

11 Gardner, D. & Shoback, D. in *Greenspan's Basic & Clinical Endocrinology: Eighth Edition* Ch. 13, 470-501 (McGraw-Hill, 2007).

12 Yoon, K., Kwack, S. J., Kim, H. S. & Lee, B. M. Estrogenic endocrine-disrupting chemicals: molecular mechanisms of actions on putative human diseases. *J. Toxicol. Environ. Health B Crit Rev* **17**, 127-174, doi:10.1080/10937404.2014.882194 (2014).

13 Colon, I., Caro, D., Bourdony, C. J. & Rosario, O. Identification of phthalate esters in the serum of young Puerto Rican girls with premature breast development. *Environ. Health Perspect* **108**, 895-900 (2000).

14 van der Meer, P. F. & Devine, D. V. Alternatives in blood operations when choosing non-DEHP bags. *Vox Sang*, doi:10.1111/vox.12479 (2017).

15 Bhat, V. S., Durham, J. L., Ball, G. L. & English, J. C. Derivation of an oral reference dose (RfD) for the nonphthalate alternative plasticizer 1,2-cyclohexane dicarboxylic acid, di-isononyl ester (DINCH). *J. Toxicol. Environ. Health B Crit Rev* **17**, 63-94, doi:10.1080/10937404.2013.876288 (2014).

16 Council, E. P. a. E. Directive 2005/84/EC of the European Parliament and of the Council of 14 December 2005 amending for the 22nd time Council Directive 76/769/EEC on the approximation of the laws, regulations and administrative provisions of the Member States relating to restrictions on the marketing and use of certain dangerous substances and preparations (phthalates in toys and childcare articles). Report No. Off.J. Eur.UnionL344, 40-43 (2005).

17 Campioli, E., Duong, T. B., Deschamps, F. & Papadopoulos, V. Cyclohexane-1,2-dicarboxylic acid diisononyl ester and metabolite effects on rat epididymal stromal vascular fraction differentiation of adipose tissue. *Environ. Res* **140**, 145-156, doi:10.1016/j.envres.2015.03.036 (2015).

18 Furr, J. R., Lambright, C. S., Wilson, V. S., Foster, P. M. & Gray, L. E., Jr. A short-term in vivo screen using fetal testosterone production, a key event in the phthalate adverse outcome pathway, to predict disruption of sexual differentiation. *Toxicol. Sci* **140**, 403-424, doi:10.1093/toxsci/kfu081 (2014).

19 Minguez-Alarcon, L. *et al.* Urinary concentrations of cyclohexane-1,2-dicarboxylic acid monohydroxy isononyl ester, a metabolite of the non-phthalate plasticizer di(isononyl)cyclohexane-1,2-dicarboxylate (DINCH), and markers of ovarian response among women attending a fertility center. *Environ. Res* **151**, 595-600, doi:10.1016/j.envres.2016.08.012 (2016).

20 Nardelli, T. C., Erythropel, H. C. & Robaire, B. Toxicogenomic Screening of Replacements for Di(2-Ethylhexyl) Phthalate (DEHP) Using the Immortalized TM4 Sertoli Cell Line. *PLoS. One* **10**, e0138421, doi:10.1371/journal.pone.0138421 (2015).

21 Eljezi, T. *et al.* In vitro cytotoxic effects of DEHP-alternative plasticizers and their primary metabolites on a L929 cell line. *Chemosphere* **173**, 452-459, doi:10.1016/j.chemosphere.2017.01.026 (2017).

22 Campioli, E. & Papadopoulos, V. Cyclohexane-1,2-dicarboxylic acid diisononyl ester and metabolite effects on rat epididymal stromal vascular fraction differentiation of adipose tissue (2015) Environmental Research 140: 145-156 Reply to the letter by Otter R. *Environ. Res* **144**, 167-169, doi:10.1016/j.envres.2015.11.002 (2016).

23 Otter, R. The publication "Cyclohexane-1,2-dicarboxylic acid diisononyl ester and metabolite effects on rat epididymal stromal vascular fraction differentiation of adipose tissue" by Enrico Campioli, Tam B. Duong, Francois Deschamps, Vassilios Papadopoulos, Environmental Research 140 (2015), 145-156, merits some critical comments. *Environ. Res* **144**, 165-166, doi:10.1016/j.envres.2015.08.021 (2016).

24 Jones, S. *et al.* Disruption of rat testis development following combined in utero exposure to the phytoestrogen genistein and antiandrogenic plasticizer di-(2-ethylhexyl) phthalate. *Biol. Reprod* **91**, 64, doi:10.1095/biolreprod.114.120907 (2014).

25 Jones, S., Boisvert, A., Francois, S., Zhang, L. & Culty, M. In utero exposure to di-(2-ethylhexyl) phthalate induces testicular effects in neonatal rats that are antagonized by genistein cotreatment. *Biol. Reprod* **93**, 92, doi:10.1095/biolreprod.115.129098 (2015).

26 Wynn, T. A. & Vannella, K. M. Macrophages in Tissue Repair, Regeneration, and Fibrosis. *Immunity* **44**, 450-462, doi:10.1016/j.immuni.2016.02.015 (2016).

27 Mendis-Handagama, S. M. & Gelber, S. J. Signs of aging are apparent in the testis interstitium of Sprague Dawley rats at 6 months of age. *Tissue Cell* **27**, 689-699 (1995).

28 McOMIE, W. A. Certain aspects of the toxicity of diallyl phthalate. *Fed. Proc* **5**, 191 (1946).

29 Dalsenter, P. R., Santana, G. M., Grande, S. W., Andrade, A. J. & Araujo, S. L. Phthalate affect the reproductive function and sexual behavior of male Wistar rats. *Hum. Exp. Toxicol* **25**, 297-303, doi:10.1191/0960327105ht624oa (2006).

30 Culty, M. *et al.* In utero exposure to di-(2-ethylhexyl) phthalate exerts both short-term and long-lasting suppressive effects on testosterone production in the rat. *Biol. Reprod* **78**, 1018-1028, doi:10.1095/biolreprod.107.065649 (2008).

31 Noriega, N. C. *et al.* Pubertal administration of DEHP delays puberty, suppresses testosterone production, and inhibits reproductive tract development in male Sprague-Dawley and Long-Evans rats. *Toxicol. Sci* **111**, 163-178, doi:10.1093/toxsci/kfp129 (2009).

32 Quinnies, K. M., Doyle, T. J., Kim, K. H. & Rissman, E. F. Transgenerational Effects of Di-(2-Ethylhexyl) Phthalate (DEHP) on Stress Hormones and Behavior. *Endocrinology* **156**, 3077-3083, doi:10.1210/EN.2015-1326 (2015).

33 Wang, C., Leung, A. & Sinha-Hikim, A. P. Reproductive aging in the male brown-Norway rat: a model for the human. *Endocrinology* **133**, 2773-2781, doi:10.1210/endo.133.6.8243304 (1993).
34 Boisvert, A. *et al.* In vitro functional screening as a means to identify new plasticizers devoid of reproductive toxicity. *Environ Res* **150**, 496-512, doi:10.1016/j.envres.2016.06.033 (2016).
35 Albert, O., Nardelli, T., Lalancette, C., Hales, B. F. & Robaire, B. in *The 9th Symposium of the Réseau Québécois en reproduction.* 83-83 (2016).
36 Chen, H., Wang, Y., Ge, R. & Zirkin, B. R. Leydig cell stem cells: Identification, proliferation and differentiation. *Mol. Cell Endocrinol*, doi:10.1016/j.mce.2016.10.010 (2016).
37 Ge, R. S. *et al.* In search of rat stem Leydig cells: identification, isolation, and lineage-specific development. *Proc. Natl. Acad. Sci. U. S. A* **103**, 2719-2724, doi:10.1073/pnas.0507692103 (2006).
38 Messam, C. A., Hou, J. & Major, E. O. Coexpression of nestin in neural and glial cells in the developing human CNS defined by a human-specific anti-nestin antibody. *Exp. Neurol* **161**, 585-596, doi:10.1006/exnr.1999.7319 (2000).
39 Jiang, M. H. *et al.* Characterization of Nestin-positive stem Leydig cells as a potential source for the treatment of testicular Leydig cell dysfunction. *Cell Res* **24**, 1466-1485, doi:10.1038/cr.2014.149 (2014).
40 Davidoff, M. S. *et al.* Progenitor cells of the testosterone-producing Leydig cells revealed. *J. Cell Biol* **167**, 935-944, doi:10.1083/jcb.200409107 (2004).
41 Liu, K., Wang, Z., Wang, H. & Zhang, Y. Nestin expression and proliferation of ependymal cells in adult rat spinal cord after injury. *Chin Med. J. (Engl.)* **115**, 339-341 (2002).
42 Schultz, R. D. *et al.* Expression of Peroxisome Proliferator-Activated Receptor alpha Messenger Ribonucleic Acid and Protein in Human and Rat Testis. *Endocrinology* **140**, 2968-2975, doi:10.1210/endo.140.7.6858 (1999).
43 Frungieri, M. B. *et al.* Number, distribution pattern, and identification of macrophages in the testes of infertile men. *Fertil. Steril* **78**, 298-306 (2002).
44 Li, X. *et al.* Transgenic mice expressing p450 aromatase as a model for male infertility associated with chronic inflammation in the testis. *Endocrinology* **147**, 1271-1277, doi:10.1210/en.2005-0654 (2006).
45 Hedger, M. P. Macrophages and the immune responsiveness of the testis. *J. Reprod. Immunol* **57**, 19-34 (2002).
46 Rigamonti, E., Chinetti-Gbaguidi, G. & Staels, B. Regulation of macrophage functions by PPAR-alpha, PPAR-gamma, and LXRs in mice and men. *Arterioscler. Thromb. Vasc. Biol* **28**, 1050-1059, doi:10.1161/ATVBAHA.107.158998 (2008).
47 Chawla, A. Control of macrophage activation and function by PPARs. *Circ. Res* **106**, 1559-1569, doi:10.1161/CIRCRESAHA.110.216523 (2010).
48 Campioli, E., Martinez-Arguelles, D. B. & Papadopoulos, V. In utero exposure to the endocrine disruptor di-(2-ethylhexyl) phthalate promotes local adipose and systemic inflammation in adult male offspring. *Nutr. Diabetes* **4**, e115, doi:10.1038/nutd.2014.13 (2014).
49 Harris, S. *et al.* The presence of macrophages and inflammatory responses in an in vitro testicular co-culture model of male reproductive development enhance relevance to in vivo conditions. *Toxicol. In Vitro* **36**, 210-215, doi:10.1016/j.tiv.2016.08.003 (2016).
50 Granholm, T., Creasy, D. M., Pollanen, P. & Soder, O. Di-n-pentyl phthalate-induced inflammatory changes in the rat testis are accompanied by local production of a novel lymphocyte activating factor. *J. Reprod. Immunol* **21**, 1-14 (1992).
51 Murphy, C. J., Stermer, A. R. & Richburg, J. H. Age- and species-dependent infiltration of macrophages into the testis of rats and mice exposed to mono-(2-Ethylhexyl) phthalate (MEHP). *Biol. Reprod* **91**, 18, doi:10.1095/biolreprod.113.115527] (2014).

52 Rajesh, P. & Balasubramanian, K. Phthalate exposure in utero causes epigenetic changes and impairs insulin signalling. *J. Endocrinol* **223**, 47-66, doi:10.1530/JOE-14-0111 (2014).

53 Zhang, W. *et al.* Di-(2-ethylhexyl) phthalate could disrupt the insulin signaling pathway in liver of SD rats and L02 cells via PPARgamma. *Toxicol. Appl. Pharmacol* **316**, 17-26, doi:10.1016/j.taap.2016.12.010 (2017).

54 Campioli, E., Batarseh, A., Li, J. & Papadopoulos, V. The endocrine disruptor mono-(2-ethylhexyl) phthalate affects the differentiation of human liposarcoma cells (SW 872). *PLoS. One* **6**, e28750, doi:10.1371/journal.pone.0028750 (2011).

Acknowledgments

We thank Mr. Charles Essagian and Ms. Annie Boisvert for assistance in handling the laboratory animals, and we thank Drs. Hanno Erythropel, Richard Leask, and Milan Maric (Department of Engineering, McGill University) for providing DINCH. Finally, we thank the Research Institute of the McGill University Health Centre Histopathology Platform and the McGill Comparative Medicine and Animal Resources Centre Laboratory for services provided. This work was supported by a grant from the Canadian Institutes of Health Research (PJT-148688), a Canada Research Chair in Biochemical Pharmacology, and the John Stauffer Dean's Chair in Pharmaceutical Sciences (to VP).

Author Contributions

Conceived and designed the experiments: EC and VP. Performed the experiments: EC, SL, ML, and LM. Analyzed the data: EC, SL, and VP. Contributed reagents/materials/analysis tools: VP. Wrote the paper: EC and VP.

Competing Financial Interests statement

The authors have no competing financial interests to declare.

Figure Legends

Figure 1. *In utero* exposure to alters gene expression in post-natal day (PND) 3 progeny without affecting fetal testosterone production. (**A**) Weight and anogenital distance (AGD) of PND 3 pups, n=14-15. (**B**) Fetal testosterone production determined by radioimmunoassay in PND 3 testes exposed *ex vivo* to control medium or medium+human chorionic gonadotropin for 1 or 2 days, n=5-6. Legend; A-b: testis A basal level, B-b: testis B basal level, A-NT: testis A not treated, B-NT: testis B treated with hCG, C: control animals, D: DINCH-treated animals. (**C**) qRT-PCR analysis of expression levels of genes involved in the steroidogenic pathway (*Lhcgr*, *Star*, *Tspo*, *Cyp11a1*), cell- and function-specific genes, including a Leydig cell marker (*Pdgfra*), a Leydig/Sertoli cell marker (*Nes*), a Sertoli cell marker (*Wt1*), a global testicular marker (*Ar*), a thyroid receptor expressed in the testes (*Thra1*), a urogenital tract marker (*Insl3*), a cell proliferation marker (*Pcna*), and germ cell markers (*Hsp90*, *Oct4*); n=9-13. Results were normalized to the β-actin gene (*Actb*) and are presented as fold-increase over control. Data in A–C are presented as the mean ± SEM. One-way analysis of variance followed by Dunnett's post hoc test was used to calculate statistical significance; *p<0.05, **p<0.01, ***p<0.001.

Figure 2. *In utero* exposure to DINCH affects reproductive health and metabolic parameters in PND 60 and PND 200 progeny. (**A**) Weight, anogenital distance (AGD), fat pad weight, seminal vesicle weight, and testes weight, n=8-10. (**B**) Random serum glucose, n=13-16. (**C**) Fasting glucose, n=4-5. (**D**) Oral glucose tolerance test; animals were gavaged with 2 g/kg of glucose, and blood was collected after 2 h, n=5. (**E**) Serum C-peptide enzyme-linked immunosorbent assay (ELISA), n=8-10. (**F**) Serum glycated hemoglobin ELISA, n=8-9. (**G**) Weight, AGD, fat pad weight, seminal vesicle weight, and testes weight, n=7-11. (**H**) Random serum glucose, n=10-13. (**I**) Fasting glucose, n=9-12. (**J**) Intraperitoneal glucose tolerance test; animals were injected intraperitoneally with 2 g/kg of glucose, and blood was collected after 2 h, n=5. (**K**) Serum C-peptide ELISA, n=9-12. (**L**) Serum glycated hemoglobin ELISA, n=9-12. (A–F) PND 60 progeny; (G–L) PND 200 progeny. Results are expressed as the mean ± SEM. One-way

analysis of variance followed by Dunnett's post hoc test or t-test (D, J) were used to calculate statistical significance; *p<0.05, **p<0.01, ***p<0.001.

Figure 3. ***In utero*** **exposure to DINCH affects circulating testosterone levels, testicular gene markers, and testicular morphology in PND 60 animals.** (**A**) qRT-PCR analysis of expression levels of genes involved in the steroidogenic pathway (*Lhcgr*, *Star*, *Tspo*, *Cyp11a1*, *Hsd3b*) and cell- and function-specific genes including a Leydig cell marker (*Pdgfra*), a Leydig/Sertoli cell marker (*Nes*), Sertoli cell markers (*Abp*, *Wt1*), a global testicular marker (*Ar*), a urogenital tract marker (*Insl3*), a cell proliferation marker (*Pcna*), and germ cell markers (*Plzf*, *Hsp90*, *Oct4*); n=9-16. (**B**) Plasma testosterone levels measured by radioimmunoassay, n=11-15. (**C**) Serum luteinizing hormone enzyme-linked immunosorbent assay (ELISA), n=7-11. (**D**) Plasma testosterone levels after a 2-week *in utero* exposure from GD 8 until parturition, n=5-6. (**E**) Period acid-Schiff (PAS) staining of PND 60 testicular tissue sections. Representative photographs from among nine animals per group; magnification 10×. (**F**) PND60 testis sections analyzed for collagen 1 immunostaining. Representative photographs from among three animals per group; magnification 10×. Gene expression results were normalized to the β-actin gene (*Actb*) and are presented as fold-increase over control (A). Data in A–D are presented as the mean ± SEM. One-way analysis of variance followed by Dunnett's post hoc test was used to calculate statistical significance; *p<0.05, **p<0.01, ***p<0.001.

Figure 4. ***In utero*** **exposure to DINCH promotes macrophage activation in PND 60 animals.** Representative images of PND60 testis sections analyzed for CD68 expression (left) and quantified macrophage infiltration (right); magnification 10×. Macrophages can be visualized as dark red spots within the tubules in the interstitial space. Data are expressed as count per number of tubules, averaged by the number of pictures (n=11) and expressed as fold change over control. The values are presented as the mean ± SEM. One-way analysis of variance followed by Dunnett's post hoc test was used to calculate statistical significance; *p<0.05, **p<0.01.

Figure 5. ***In utero*** **exposure to DINCH alters circulating testosterone without affecting macrophage infiltration in PND200 animals.** (**A**) qRT-PCR analysis of expression levels of genes involved in the steroidogenic pathway (*Lhcgr*, *Star*, *Tspo*, *Cyp11a1*, *Hsd3b*) and cell- and function-specific genes, including a Leydig cell marker (*Pdgfra*), a Sertoli cell marker (*Wt1*), a global testicular marker (*Ar*), a cell proliferation marker (*Pcna*), and germ cell markers (*Plzf*, *Hsp90*, *Oct4*); n=9-12. (**B**) Plasma testosterone levels measured by radioimmunoassay, n=10-13. (**C**) Serum luteinizing hormone enzyme-linked immunosorbent assay, n=7-10. (**D**) PAS staining of PND 200 testicular tissue sections. Representative photographs from among nine animals per group; magnification 10×. (**E**) Representative images of PND200 testis sections analyzed for CD68 expression (left) and quantified macrophage infiltration (right); magnification 10×. Macrophages can be visualized as dark red spots within the tubules in the interstitial space. Data are expressed as count per number of tubules, averaged by the number of pictures (n=11) and expressed as fold change over control. Gene expression results were normalized to the β-actin gene (*Actb*) and are presented as fold-increase over control (A). Data in A–C are presented as the mean ± SEM. One-way analysis of variance followed by Dunnett's post hoc test was used to calculate statistical significance; *p<0.05, **p<0.01, ***p<0.001.

Figure 6. In utero exposure to 100mg DINCH/kg/daqy promotes the occurrence of testicular atrophy in PND 200 animals. (**A**) Images of normal and atrophied testes containing degenerating seminiferous tubules and the rate of occurrence in offspring exposed *in utero* to 100 mg DINCH/kg/day. (**B**) Representative images of PAS staining of sections from normal and atrophied testes containing degenerating tubules; magnification 10×. (**C**) Representative images of sections analyzed for CD68 expression in normal and atrophied testes containing degenerating tubules; magnification 10×.

Table 1. Analysis of blood from PND 60 progeny.

		Control (n=16)	DINCH 1 mg (n=15)	DINCH 10 mg (n=13)	DINCH 100 mg (n=16)	Significance
KIDNEY	*BUN Urea (mmol/L)*	5.77 ± 0.21	5.35 ± 0.19	5.72 ± 0.21	5.31 ± 0.14	n.s
	Creatinine (µmol/L)	21.25 ± 1.25	24.00 ± 1.19	18.92 ± 1.86	21.63 ± 0.95	n.s
	Calcium (mmol/L)	3.11 ± 0.04	3.22 ± 0.05	3.07 ± 0.06	3.10 ± 0.05	n.s.
	Chloride (mmol/L)	103.50 ± 2.28	101.10 ± 0.61	101.50 ± 0.76	100.90 ± 0.44	n.s.
	Magnesium (mmol/L)	1.69 ± 0.05	1.79 ± 0.04	1.54 ± 0.06	1.43 ± 0.04***	p<0.0001
	Phosphorus (mmol/L)	4.29 ± 0.16	4.23 ± 0.12	4.31 ± 0.22	4.05 ± 0.12	n.s.
	Potassium (mmol/L)	7.66 ± 0.52	7.13 ± 0.29	7.85 ± 0.53	6.81 ± 0.29	n.s.
	Sodium (mmol/L)	150.80 ± 2.23	149.50 ± 2.48	146.10 ± 0.66	146.90 ± 0.47	n.s.
LIVER	*Albumin (g/L)*	38.31 ± 1.37	35.73 ± 0.32	37.46 ± 1.04	36.56 ± 0.92	n.s.
	Albumin/Globulin ratio	1.07 ± 0.02	1.16 ± 0.02	1.17 ± 0.05	1.21 ± 0.03**	p=0.0120
	Alkaline Phosphatase (U/L)	311.50 ± 15.28	311.70 ± 19.16	307.80 ± 15.81	293.40 ± 13.29	n.s.
	ALT (U/L)	53.36 ± 2.98	50.29 ± 2.53	54.25 ± 2.51	53.77 ± 4.77	n.s.
	AST (U/L)	184.30 ± 23.90	120.70 ± 9.81	178.10 ± 26.54	141.10 ± 20.61	n.s.
	Total Bilirubin (mmol/L)	12.64 ± 0.83	8.00 ± 0.44***	10.86 ± 1.47	8.25 ± 1.01**	p=0.0009
	Total Protein (g/L)	73.50 ± 2.47	66.67 ± 0.75*	69.38 ± 1.65	67.56 ± 1.54*	p=0.0320
LIPIDS	*Cholesterol (mmol/L)*	2.43 ± 0.09	2.39 ± 0.10	2.22 ± 0.10	2.40 ± 0.08	n.s.
	HDL (mmol/L)	1.48 ± 0.05	1.56 ± 0.06	1.37 ± 0.05	1.51 ± 0.05	n.s.
	Triglycerides (mmol/L)	2.93 ± 0.20	2.15 ± 0.13*	2.80 ± 0.27	2.46 ± 0.21	p=0.043

ALT, alanine aminotransferase; AST, aspartate aminotransferase; BUN, Blood urea nitrogen; DINCH, 1,2-cyclohexane dicarboxylic acid diisononyl ester; HDL, High-density lipoproteins. One-way analysis of variance followed by Dunnett's post hoc test was used to calculate statistical significance, *p<0.05, **p<0.01, ***p<0.001, *n*.s, non significant.

Table 2. Analysis of blood from PND 200 progeny.

		Control (N=11)	*DINCH 1 mg (N=13)*	*DINCH 10 mg (N=10)*	*DINCH 100 mg (N=13)*	*Significance*
KIDNEY	*BUN Urea (mmol/L)*	6.53 ± 0.22	6.35 ± 0.20	6.08 ± 0.21	5.98 ± 0.21	n.s.
	Creatinine (μmol/L)	30.27 ± 1.56	30.54 ± 1.78	29.90 ± 2.14	28.54 ± 1.18	n.s.
	Calcium (mmol/L)	3.01 ± 0.04	3.06 ± 0.05	3.05 ± 0.05	3.00 ± 0.04	n.s.
	Chloride (mmol/L)	100.10 ± 1.01	101.10 ± 0.59	101.10 ± 0.57	100.30 ± 0.43	n.s.
	Magnesium (mmol/L)	1.64 ± 0.07	1.67 ± 0.07	1.68 ± 0.07	1.54 ± 0.06	n.s.
	Phosphorus (mmol/L)	3.95 ± 0.13	4.05 ± 0.21	3.82 ± 0.10	3.57 ± 0.10	n.s.
	Potassium (mmol/L)	8.57 ± 0.38	8.79 ± 0.51	9.18 ± 0.57	7.81 ± 0.21	n.s.
	Sodium (mmol/L)	147.50 ± 0.56	148.70 ± 0.52	147.80 ± 1.09	147.30 ± 0.86	n.s.
LIVER	*Albumin (g/L)*	40.27 ± 1.00	39.31 ± 0.84	41.60 ± 1.19	39.46 ± 0.68	n.s.
	Albumin/Globulin ratio	1.31 ± 0.04	1.29 ± 0.04	1.33 ± 0.05	1.30 ± 0.04	n.s.
	Alkaline Phosphatase (U/L)	164.20 ± 11.39	141.60 ± 6.40	157.00 ± 13.42	153.0 ± 11.49	n.s.
	ALT (U/L)	78.70 ± 10.92	80.82 ± 12.62	113.10 ± 25.52	172.80 ± 58.22	n.s.
	AST (U/L)	229.80 ± 49.17	204.10 ± 46.10	264.10 ± 55.71	421.30 ± 125.80	n.s.
	Total Bilirubin (mmol/L)	12.20 ± 1.07	10.82 ±	12.71 ± 2.90	10.92 ± 1.55	n.s.
	Total Protein (g/L)	71.18 ± 0.88	70.38 ± 1.60	73.20 ± 1.11	69.92 ± 0.88	n.s.
LIPIDS	*Cholesterol (mmol/L)*	3.15 ± 0.18	2.84 ± 0.14	2.75 ± 0.20	2.91 ± 0.18	n.s.
	HDL (mmol/L)	1.80 ± 0.07	1.61 ± 0.07	1.62 ± 0.08	1.73 ± 0.10	n.s.
	Triglycerides (mmol/L)	2.97 ± 0.30	2.77 ± 0.24	2.70 ± 0.35	2.70 ± 0.17	n.s.

ALT, alanine aminotransferase; AST, aspartate aminotransferase; BUN, Blood urea nitrogen; DINCH, 1,2-cyclohexane dicarboxylic acid diisononyl ester; HDL, High-density lipoproteins. One-way analysis of variance followed by Dunnett's post hoc test was used to calculate statistical significance, *n*.s, non significant.

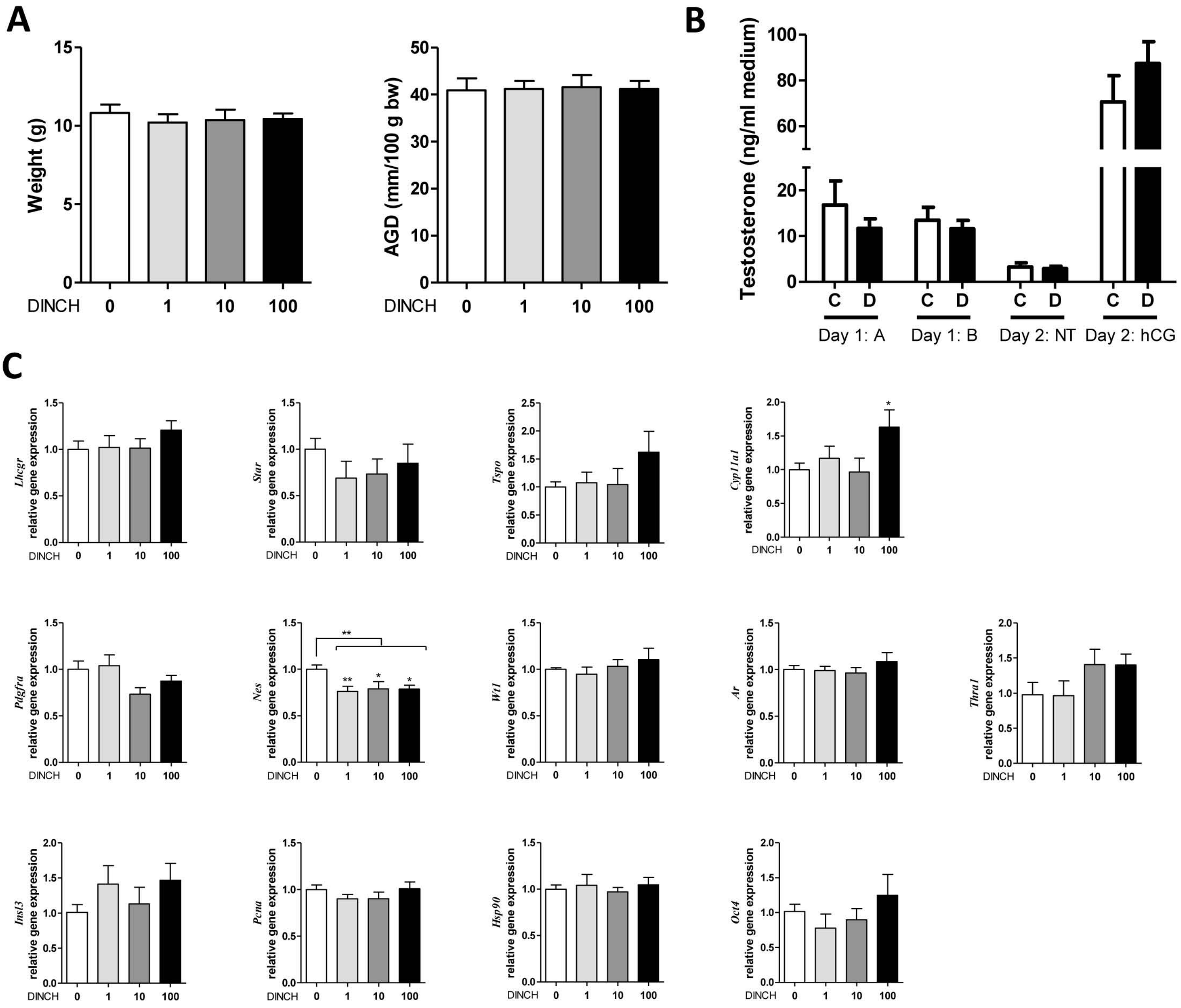

A
Weight (g)
AGD (mm/100 g bw)
DINCH
0
1
10
100
B
Testosterone (ng/ml medium)
C
D
Day 1: A
Day 1: B
Day 2: NT
Day 2: hCG
C
Lhcgr
Star
Tspo
Cyp11a1
Pdgfra
Nes
Wt1
Ar
Thra1
Insl3
Pcna
Hsp90
Oct4
relative gene expression

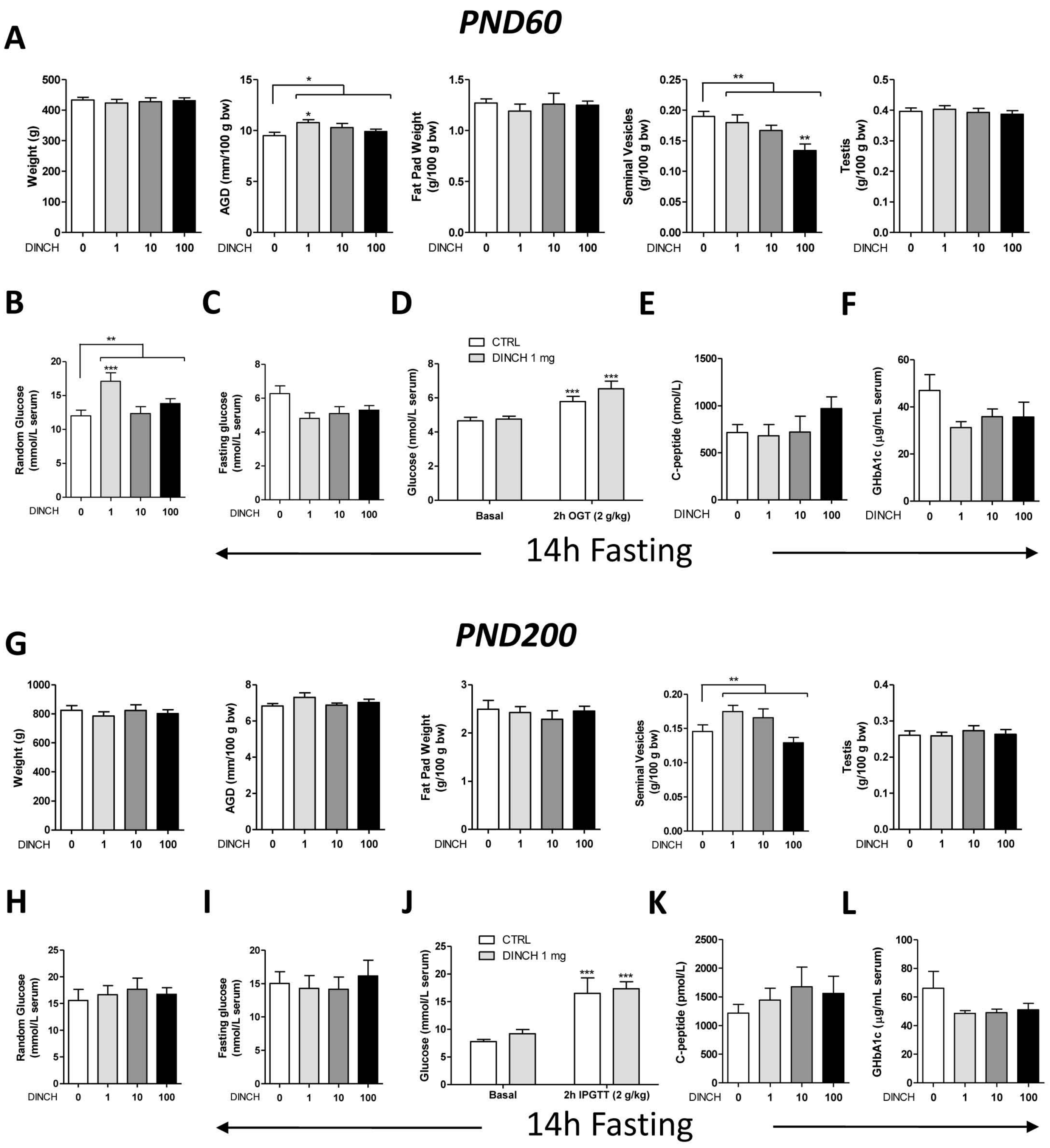
PND60
A
Weight (g)
AGD (mm/100 g bw)
Fat Pad Weight (g/100 g bw)
Seminal Vesicles (g/100 g bw)
Testis (g/100 g bw)
DINCH 0 1 10 100
B
C
D
E
F
Random Glucose (mmol/L serum)
Fasting glucose (nmol/L serum)
Glucose (nmol/L serum)
CTRL
DINCH 1 mg
Basal
2h OGT (2 g/kg)
C-peptide (pmol/L)
GHbA1c (µg/mL serum)
14h Fasting
PND200
G
H
I
J
K
L
Random Glucose (mmol/L serum)
Fasting glucose (nmol/L serum)
Glucose (mmol/L serum)
Basal
2h IPGTT (2 g/kg)
C-peptide (pmol/L)
GHbA1c (µg/mL serum)
14h Fasting

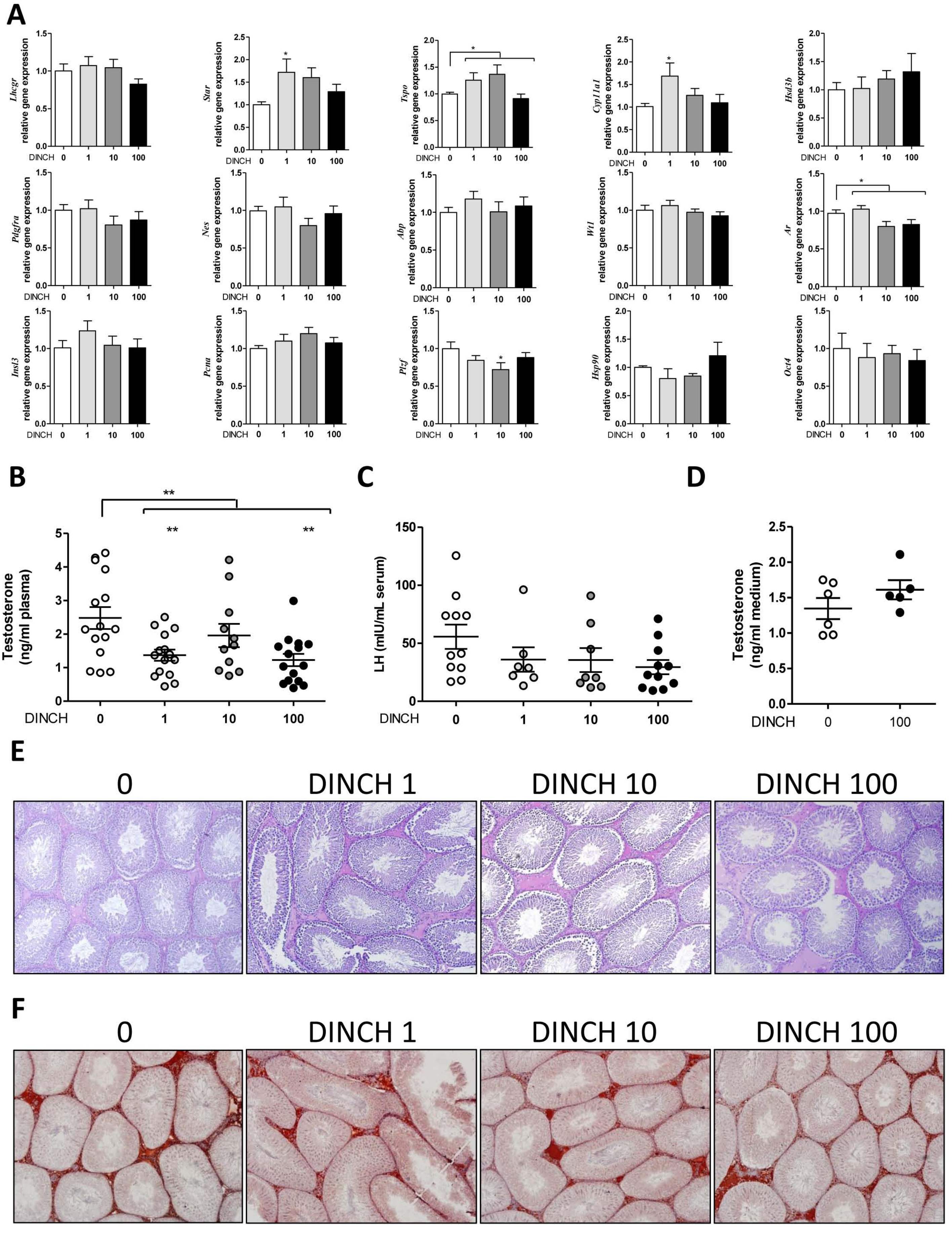
A
Lhcgr relative gene expression
Star relative gene expression
Tspo relative gene expression
Cyp11a1 relative gene expression
Hsd3b relative gene expression
Pdgfra relative gene expression
Nes relative gene expression
Abp relative gene expression
Wt1 relative gene expression
Ar relative gene expression
Insl3 relative gene expression
Pcna relative gene expression
Plzf relative gene expression
Hsp90 relative gene expression
Oct4 relative gene expression
DINCH 0 1 10 100
B
Testosterone (ng/ml plasma)
C
LH (mIU/mL serum)
D
Testosterone (ng/ml medium)
E
0
DINCH 1
DINCH 10
DINCH 100
F
0
DINCH 1
DINCH 10
DINCH 100

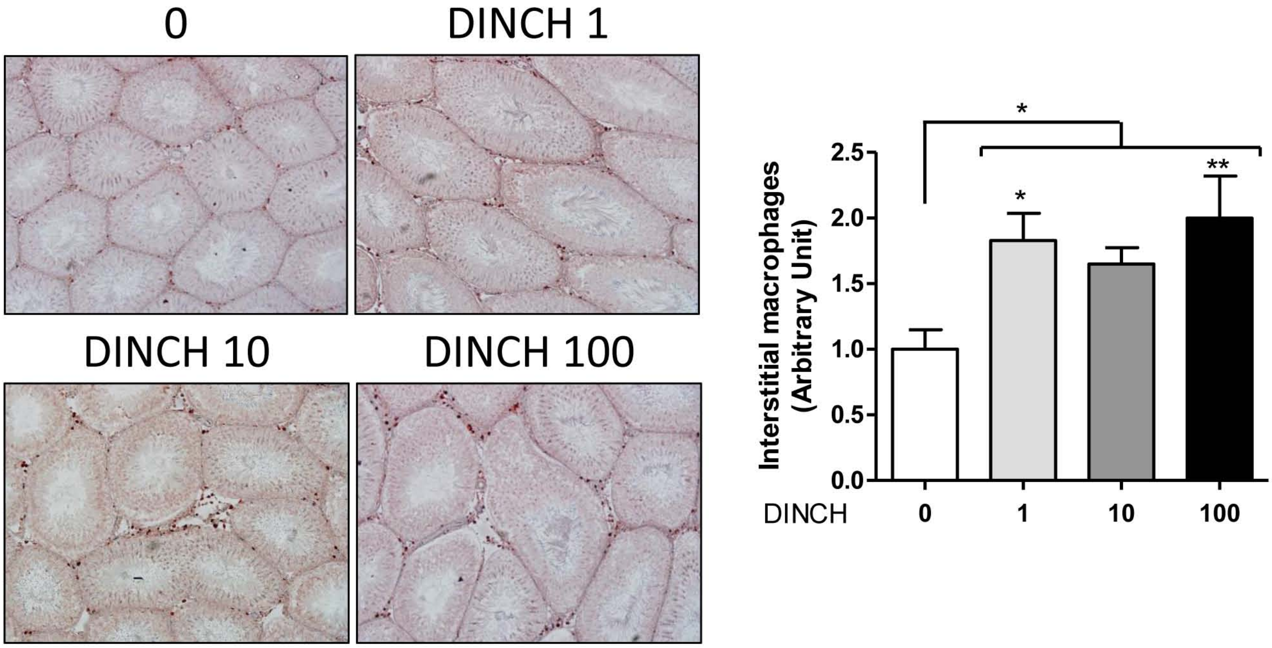
0
DINCH 1
DINCH 10
DINCH 100
Interstitial macrophages
(Arbitrary Unit)
2.5
2.0
1.5
1.0
0.5
0.0
*
*
**
DINCH
0
1
10
100

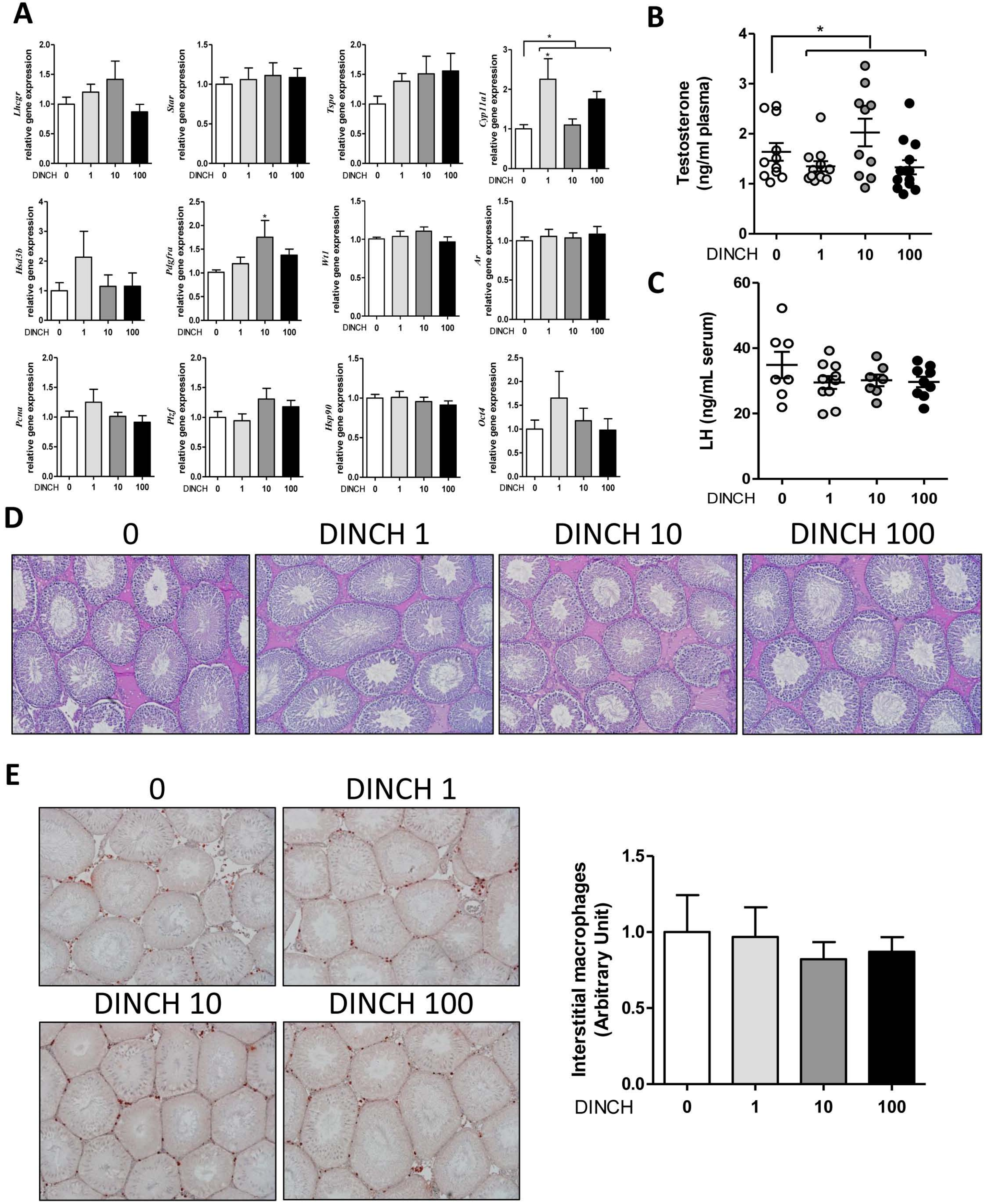
A
Lhcgr relative gene expression
Star relative gene expression
Tspo relative gene expression
Cyp11a1 relative gene expression
Hsd3b relative gene expression
Pdgfra relative gene expression
Wt1 relative gene expression
Ar relative gene expression
Pcna relative gene expression
Plzf relative gene expression
Hsp90 relative gene expression
Oct4 relative gene expression
DINCH 0 1 10 100
B
Testosterone (ng/ml plasma)
DINCH 0 1 10 100
C
LH (ng/mL serum)
DINCH 0 1 10 100
D
0
DINCH 1
DINCH 10
DINCH 100
E
0
DINCH 1
DINCH 10
DINCH 100
Interstitial macrophages (Arbitrary Unit)
DINCH 0 1 10 100

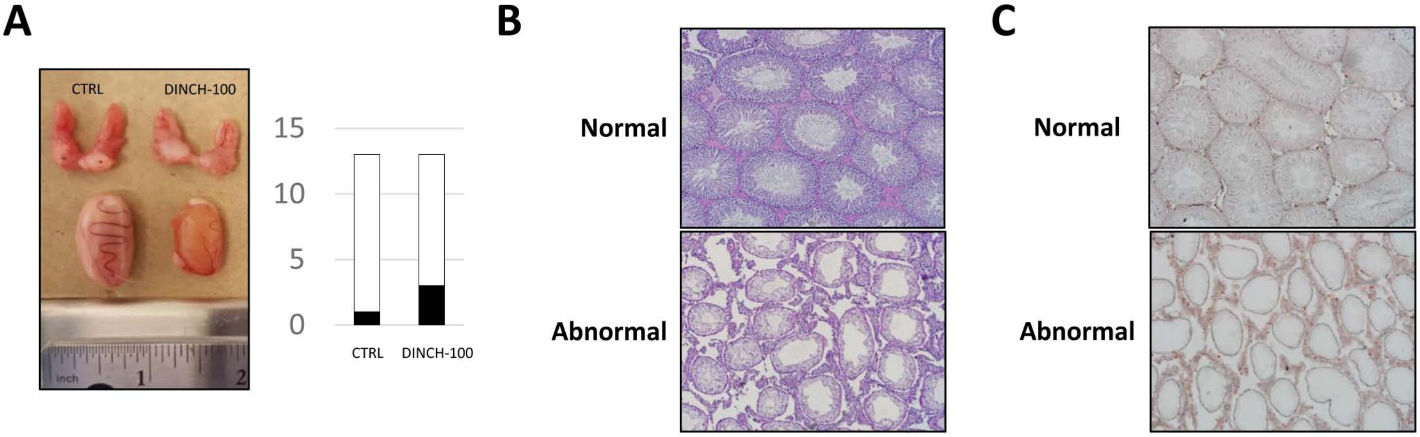
A
CTRL
DINCH-100
inch
1
2
15
10
5
0
CTRL
DINCH-100
B
Normal
Abnormal
C
Normal
Abnormal
